# Novel Medical Treatments and Devices for the Management of Heart Failure with Reduced Ejection Fraction

**DOI:** 10.3390/jcdd11040125

**Published:** 2024-04-19

**Authors:** Michele Alfieri, Filippo Bruscoli, Luca Di Vito, Federico Di Giusto, Giancarla Scalone, Procolo Marchese, Domenico Delfino, Simona Silenzi, Milena Martoni, Federico Guerra, Pierfrancesco Grossi

**Affiliations:** 1 Cardiology and Arrhythmology Clinic, Marche Polytechnic University, University Hospital “Umberto I-Lancisi-Salesi”, 60121 Ancona, Italy; m.alfieri95@gmail.com (M.A.); f.guerra@univpm.com (F.G.); 2 Cardiology Unit, C. and G. Mazzoni Hospital, AST Ascoli Piceno, 63100 Ascoli Piceno, Italy; filippo.bruscoli@gmail.com (F.B.); digiusto.federico@gmail.com (F.D.G.); gcarlascl@gmail.com (G.S.); procolo.marchese@gmail.com (P.M.); delfino.domenico31@gmail.com (D.D.); silenzi.s@libero.it (S.S.); p.f.grossi@gmail.com (P.G.); 3 Medical School, Università degli Studi “G. d’Annunzio”, 66100 Chieti, Italy; milena.martoni@gmail.com

**Keywords:** heart failure, reduced ejection fraction, SGLT2 inhibitors, vericiguat, CardioMEMS, cardiac contractility modulation, left bundle branch area pacing, wearable cardioverter defibrillators, ultrafiltration

## Abstract

Heart failure (HF) is a growing issue in developed countries; it is often the result of underlying processes such as ischemia, hypertension, infiltrative diseases or even genetic abnormalities. The great majority of the affected patients present a reduced ejection fraction (≤40%), thereby falling under the name of “heart failure with reduced ejection fraction” (HFrEF). This condition represents a major threat for patients: it significantly affects life quality and carries an enormous burden on the whole healthcare system due to its high management costs. In the last decade, new medical treatments and devices have been developed in order to reduce HF hospitalizations and improve prognosis while reducing the overall mortality rate. Pharmacological therapy has significantly changed our perspective of this disease thanks to its ability of restoring ventricular function and reducing symptom severity, even in some dramatic contexts with an extensively diseased myocardium. Notably, medical therapy can sometimes be ineffective, and a tailored integration with device technologies is of pivotal importance. Not by chance, in recent years, cardiac implantable devices witnessed a significant improvement, thereby providing an irreplaceable resource for the management of HF. Some devices have the ability of assessing (CardioMEMS) or treating (ultrafiltration) fluid retention, while others recognize and treat life-threatening arrhythmias, even for a limited time frame (wearable cardioverter defibrillator). The present review article gives a comprehensive overview of the most recent and important findings that need to be considered in patients affected by HFrEF. Both novel medical treatments and devices are presented and discussed.

## 1. Introduction

Heart failure (HF) is a common condition currently defined as a clinical syndrome made of symptoms and signs and characterized by structural and/or functional cardiac abnormalities leading to an inadequate oxygen supply or elevated intracavitary pressures [1].

In this population, the majority of patients are represented by the class of HF with reduced ejection fraction (HFrEF). In these patients, the inability of maintaining an adequate peripheral perfusion induces the activation of neurohormonal pathways which, in the long term, lead to cardiac remodeling and increased afterload, thereby exacerbating the underlying dysfunction [2]. Patients with HFrEF are also at an increased risk of arrhythmic events favored by neurohormonal activation, myocyte dysfunction and, in some contexts, by scar formation. This risk may, in some instances, be temporary due to the potential improvement in heart function through neurohormonal modulation and afterload reduction [3].

In recent years, new drugs have been developed, and their capability of acting on different pathways provides complementary benefits; in order to properly express such effectiveness, these treatments should be initiated early and quickly titrated.

Along with pharmacological progression, new technologies have been developed in order to provide additional treatments able to act on the natural course of the disease together with a multiparametric monitoring of HF patients.

The review article is divided into two main chapters: the first one is focused on new medical treatments for HFrEF and different strategies for their application on “naïve” patients, while the second one is targeted on new devices for both monitoring and treating advanced HFrEF patients.

## 2. New Medical Treatments for HFrEF

### 2.1. Sodium–Glucose Transport Protein 2 Inhibitors

Sodium–glucose transport protein 2 (SGLT2) inhibitors, also called gliflozins, are a class of drugs that inhibit SGLT2 in the proximal convoluted tubule of nephron. The main metabolic effect is to inhibit the reabsorption of glucose in the kidney and subsequently lower blood sugar. They were initially prescribed as oral hypoglycemic agents, and they are currently used for the treatment of type 2 diabetes mellitus. The investigators of earlier SGLT2 inhibitor studies found that they had exceptional cardiovascular benefits in patients with diabetes mellitus [4,5,6]. The precise mechanisms through which SGLT2 inhibitors exert their positive action in HF are not completely understood, and some hypotheses have been raised:

- *Blood pressure reduction:* An increase in urine glucose excretion as well as reduced sodium reabsorption have been proposed to explain the reducing effect on blood pressure [7]. SGLT2 inhibitors impacted weight loss [8]. They also act on cholesterol levels by showing a low increase in HDL, LDL and total cholesterol levels and a small decrease in triglycerides [9]. However, the marked benefit seen with the use of these drugs in patients with HFrEF is not completely explained by the improvement of the cardiovascular risk factors [10]. It has been hypothesized that other mechanisms could contribute to the beneficial effects observed in HF patients:
- *Effect on cardiac remodeling and contractility:* Previous studies have demonstrated that empagliflozin led to a significant reduction in diastolic tension without altering the systolic contractile force [11,12]. Empagliflozin decreases myofilament stiffness in human myocardium through an enhanced phosphorylation process of titin due to the improvement of the nitric oxide (NO) pathway [11].
- *Reduction in inflammation:* Several studies showed that SGLT2 inhibitors have anti-inflammatory and antioxidant properties [13]. Dapagliflozin reduced the inflammasome and fibrosis in mouse models of type 2 diabetes mellitus and myocardial infarction [14], and empagliflozin decreased oxidative stress, associated with metabolic changes, in mice after myocardial infarction [15].
- *Cardiorenal function improvement:* SGLT2 inhibitors cause a natriuretic effect, favoring sodium excretion by the distal renal tubules, inducing a vasoconstriction of the afferent arteriolar vessel and finally restoring the impaired tubuloglomerular feedback mechanism [16]. As a result, the reduced blood flow elicits the release of erythropoietin [17].

Based on these positive effects on the cardiovascular system, SGLT2 inhibitors are currently recommended for patients with HFrEF.

The EMPA-REG OUTCOME trial evaluated cardiovascular outcomes with empagliflozin in patients with type 2 diabetes mellitus and with established cardiovascular disease. The treatment with SGLT2 inhibitors significantly reduced the primary composite endpoint of major adverse cardiovascular events (cardiovascular mortality, nonfatal stroke, nonfatal myocardial infarction and major cardiovascular adverse events) compared to the placebo, primarily attributed to a 38% significant reduction in cardiovascular death. Empagliflozin treatment was able to reduce HF hospitalizations by 35% [4]. The Canagliflozin and Cardiovascular and Renal Events in Type 2 Diabetes (CANVAS) [5] and the Dapagliflozin and Cardiovascular Outcomes in Type 2 Diabetes (DECLARE TIMI) [6] trials found similar results with canagliflozin and dapagliflozin, respectively.

Other studies were conducted on the role of SGLT2 inhibitors in the management of HF. The Dapagliflozin and Prevention of Adverse Outcomes in Heart Failure (DAPA-HF) [18] trial was the first study that evaluated the efficacy of an SGLT-2 inhibitor in 4744 patients with stable chronic HFrEF (LVEF < 40%). Dapagliflozin was found to significantly reduce the primary composite outcome of cardiovascular death, HF hospitalizations and urgent HF visits compared to the placebo. Of note, the results were identified independently of baseline diabetes mellitus status. A subanalysis of dapagliflozin trials conducted to identify the impact of dapagliflozin across death types showed that this drug significantly reduced the rate of sudden and HF deaths. A lower impact was shown for death due to stroke or myocardial infarction [19]. Similar results were found for empagliflozin [20].

Another study, named the Empagliflozin Outcome Trial in Patients with Chronic Heart Failure and a Reduced Ejection Fraction (EMPEROR-Reduced) [21] evaluated the efficacy of empagliflozin in a similar cohort of 3730 HF patients. Empagliflozin significantly reduced the composite outcome of cardiovascular death and HF hospitalizations compared to the placebo, with a relative risk reduction of 21%. The results were driven by a significant 31% relative risk reduction in HF hospitalizations.

Given the considerable results of the DAPA-HF and EMPEROR-Reduced trials, the SGLT2 inhibitors have become one of the main treatments for heart failure. The European Society of Cardiology (ESC) 2021 HF guidelines [22] and the American Heart Association/American College of Cardiology/Heart Failure Society of America (AHA/ACC/HFSA) 2022 HF guidelines [23] recommend the use of SGLT-2 inhibitors (currently only dapagliflozin and empagliflozin) in the treatment of chronic, stable HFrEF with a class 1 recommendation for a reduction in cardiovascular death and HF hospitalizations irrespective of baseline diabetes mellitus (Table 1).

### 2.2. Vericiguat

One of the principal mechanisms involved in HF is the reduced amount of NO or the resistance to this molecule [24,25]. NO acts directly via the stimulation of the soluble guanylate cyclase (sGC), which produces cyclic guanosine monophosphate, which in turn contributes to the maintenance of vascular tone and cardiac contractility. Other effects include the reduction in profibrotic and inflammatory pathways and the counteraction of myocyte hypertrophy [26,27].

Vericiguat was the first sGC stimulator approved for HF on the market and acts both by directly stimulating sGC and by increasing its sensitivity to NO [28].

The principal study that applied this drug on top of optimized medical therapy (OMT) is the VICTORIA study that showed a significant reduction in the primary outcome of first hospitalization for HF or death from cardiovascular causes. Secondary outcomes of total hospitalizations for HF and death from any cause or first hospitalization for HF were also significantly reduced. The study included patients in New York Heart Association (NYHA) class II-IV, left ventricular ejection fraction (LVEF) < 45%, recent (under six months) hospitalization for HF or intravenous diuretics administration in the previous three months, high levels of natriuretic peptides (in sinus rhythm BNP ≥ 300 pg/mL or NT-proBNP ≥ 1000 pg/mL, in atrial fibrillation BNP ≥ 500 pg/mL or NT-proBNP ≥ 1600 pg/mL) and an estimated glomerular filtration rate (eGFR) ≥ 15 mL/min. The benefit was independent of the ongoing treatment and was more evident in patients with NT-proBNP ≤ 4000 pg/mL. Of note, a significant benefit was not found for NT-proBNP values > 8000 pg/mL. Symptomatic hypotension, syncope and the principal side effects were not different among groups [25,29].

**Figure 1:**
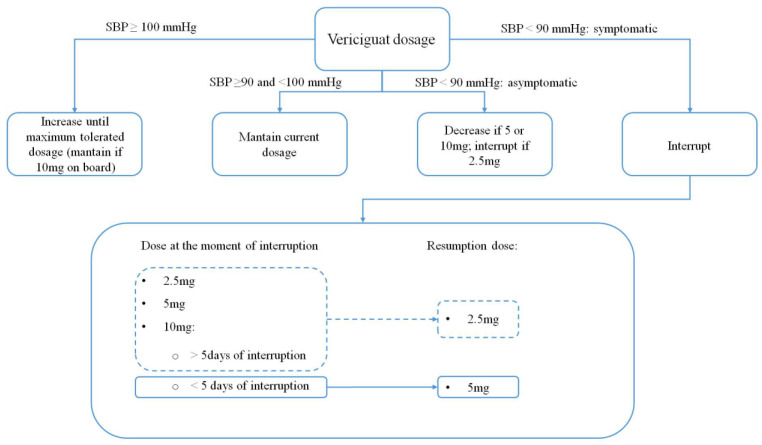
Vericiguat dose modifications according to VICTORIA trial protocol.

Hypotension may require a temporary down-titration or even an interruption of the drug. Data from the trial protocol give precise recommendations regarding the correct management according to symptoms and blood pressure values [29] (Figure 1).

Despite the benefits shown in hospitalizations and mortality, the effects of vericiguat on the heart are still largely unknown. An echocardiographic substudy [30] of the VICTORIA trial was conducted but failed to identify an improvement in LVEF or other echocardiographic parameters compared to the placebo group. The current 2021 ESC guidelines give vericiguat a recommendation IIb, level of evidence B, for symptomatic patients (NYHA II-IV) with reduced LVEF and worsening HF despite OMT to reduce cardiovascular mortality and hospitalizations [31].

The AHA/ACC/HFSA 2022 guidelines give a class 2b recommendation for this drug once OMT is achieved and the patient remain in NYHA II-IV, with LVEF < 45%, recent heart failure hospitalization or intravenous diuretic therapy and elevated natriuretic peptide levels [23].

The starting dose of 2.5 mg can be doubled every two weeks to reach the target dose of 10 mg. Its bioavailability is high when taken with food (93%), and the plasma half-life reaches 30 h in patients with HF [27]. No significant drug–drug interactions have been detected, although proton pump inhibitors may reduce its absorption, and the association with phosphodiesterase 5 inhibitors, long-acting nitrates or other sGCS like riociguat should be avoided [28].

Given the reduction in blood pressure values (of approximately 1–2 mmHg) and the potential detrimental effects on hemoglobin levels, it should not be prescribed in patients with systolic blood pressure < 100 mmHg or anemia; further contraindications are pregnancy and severe liver impairment [28,29].

### 2.3. Pharmacological Treatment Strategies for Heart Failure with Reduced Ejection Fraction

Pharmacological treatment is the mainstay of HFrEF therapy and should be calibrated and up-titrated before considering device therapy.

The three main objectives of HFrEF therapy are as follows: (1) a reduction in mortality, (2) the prevention of recurrent hospitalizations due to worsening HF, and (3) an improvement in quality of life [32].

In patients with symptomatic HFrEF on OMT, the administration of an angiotensin receptor neprilysin inhibitor (ARNI) is recommended instead of an ACE inhibitor or ARB to further reduce mortality and morbidity. In fact, the PARADIGM-HF trial showed the superiority of ARNI for both cardiovascular death and HF hospitalizations [33].

In addition, SGLT2 inhibitors, dapagliflozin or empagliflozin, are recommended independently from the presence of diabetes mellitus to further decrease the risk of worsening HF or cardiovascular death. The ESC guidelines also recommend the use of ARNI as a substitution for an ACE inhibitor in patients who remain symptomatic on ACE inhibitors, β-blockers and MRA. However, ARNI may be considered as a first-line therapy instead of an ACE inhibitor/ARB in patients with a severely reduced LVEF for whom a complete recovery of LV function is improbable to achieve [22]. Diuretics are usually required to improve symptoms due to fluid overload in the decompensated phase of HF. However, there is no evidence of their effect on survival.

Ivabradine, a selective inhibitor of If current, may be considered to reduce the risk of HF hospitalization or cardiovascular death in patients with symptomatic HFrEF with a sinus rhythm and heart rate of 70–75 bpm or higher despite OMT [34].

**Figure 2:**
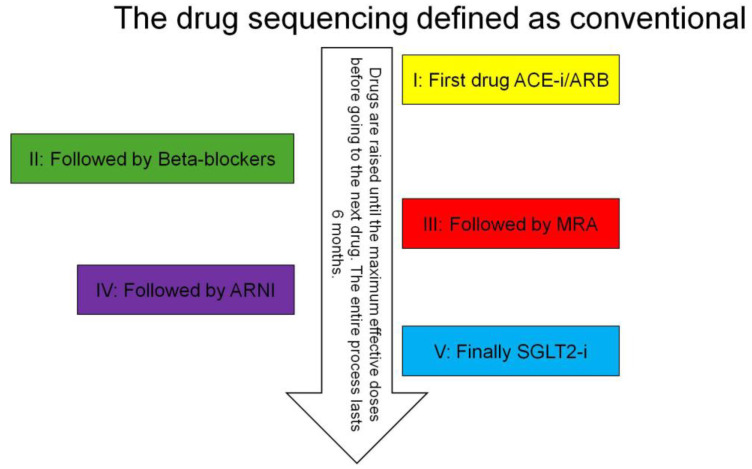
Conventional sequencing add-on therapy for patient with HFrEF [36].

**Figure 3:**
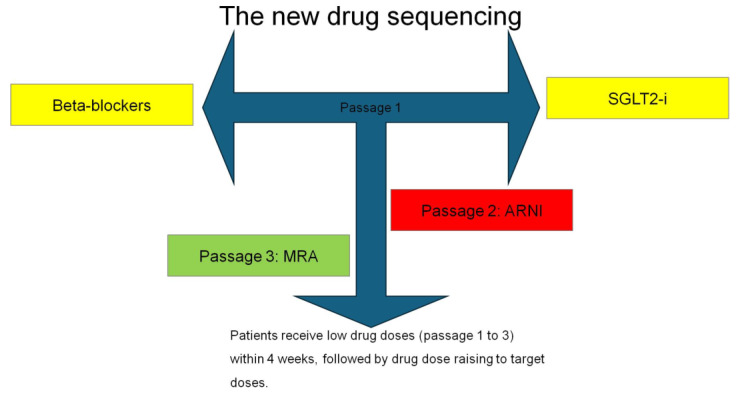
New sequencing add-on therapy for patient with HFrEF [36].

As mentioned previously, the *conventional sequencing* strategy of OMT for chronic HFrEF recommends starting the treatment with ACE inhibitors or ARBs, β-blockers and MRA. In the second phase, ARNI can be administered instead of ACEI/ARB and SGLT2 inhibitors added to the medical treatment (Figure 2). Unfortunately, this step-by step approach may not be coherent with the fact that the most effective therapeutic approaches should be started as early as possible [35]. Thus, McMurray et al. proposed a *new sequencing* strategy instead of the traditional approach [36]. Subsequently, other authors adopted the new sequencing strategy [37,38]. The revised approach requires the early initiation of all the major four classes of HF drugs (ARNI, β-blockers, MRA and SGLT2 inhibitors) [36] at their lower dosages (Figure 3). This revised approach can be particularly efficient if started in hospitalized patients before hospital discharge to verify patient compliance and side effect occurrences.

The principles that support the revised strategy are as follows: First, the level of benefit obtained with any class of drugs is independent of that produced by other classes. Second, it has been demonstrated that low initiating regimens of principal drugs are successful in reducing morbidity and mortality. Third, the introduction of a new drug class leads to benefits that are greater than the up-titration of ongoing drug classes. Fourth, because a great part of the benefits of principal medications are observed within the 30 days of starting treatment, the strategy should entail starting with all four types of drugs within the first month [36].

The revised approach requires three steps as follows:

Step 1: This consists of a coincident initiation of treatment with a β-blocker and an SGLT2 inhibitor. Indeed, β-blockers are the drug class with the largest evidence for reducing the risk of sudden death. SGLT2 inhibitors have a high effectiveness for reducing the risk of hospitalizations for HF. SGLT2 inhibitors, due to their diuretic action, may alleviate the short-term risk of worsening HF that may occur after a β-blocker has started.

Step 2: This includes the addition of ARNI/ACEI/ARB within 1 to 2 weeks of step 1. In case of an early initiation of ARNI, if the patient’s systolic blood pressure is low (<100 mm Hg), it may be useful to first assess hypotension tolerance, with an ARB before switching to ARNI. Usually, hypotension is commonly resolved with reducing the dose or with an adjustment of the dose of concurrently administered diuretics.

Step 3: This consists of an addition of an MRA within 1 to 2 weeks of step 2. Step 3 should be preceded by checking the normality of serum potassium and renal function. In this setting, the positive effects of ARNI and SGLT2 inhibitors on renal function and potassium homeostasis may favor an MRA prescription [36].

**Figure 4:**
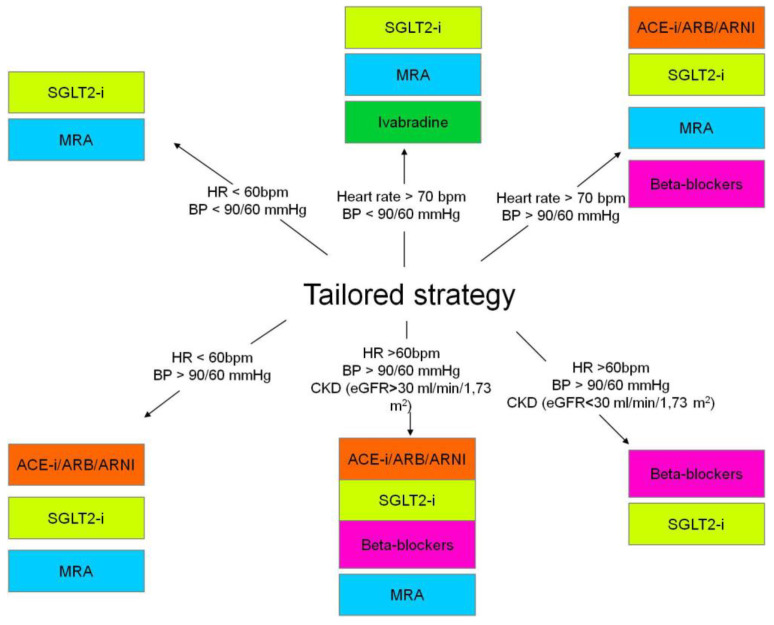
The tailored strategy depending on patient clinical features. Heart rate (HR), blood pressure (BP) and chronic kidney disease (CKD).

The last proposed pharmacological strategy is the *tailored* one, described in a consensus document of the ESC [39]. The *tailored strategy* requires one to choose pharmacological therapy in HFrEF based on the patient profile. This strategy adjusts medical therapies to the hemodynamic parameters, considering signs of congestion and kidney function together with blood pressure and heart rate [39] (Figure 4).

## 3. New Devices for Monitor or Treat HFrEF Patients

### 3.1. Cardio-Microelectromechanical Systems

Although many innovations in drug treatment for HF have been introduced, HF patients still require frequent ambulatory visits, and they often require hospitalization for acute decompensated HF. These hospital admissions usually last several days and require large amounts of healthcare resources. Repeated hospital admissions for decompensated HF are associated with a decline in myocardial and renal function and additionally can worsen survival [40]. Thus, one of the targets of HF management is the reduction in HF hospitalizations.

Towards this end, in the early 2000s, the cardio-microelectromechanical system (MEMS) was proposed. CardioMEMS is a wireless pressure sensor that uses MEMS technology. This device consists of an implantable HF sensor and an electronic monitoring unit. It is implanted by right heart catheterization in the distal pulmonary artery. The main role of the sensor is to measure changes in pulmonary artery pressure (PAP). CardioMEMS does not have any batteries or leads, and it is powered by an external antenna in the form of radiofrequency signals. Patients use an electronic unit and a special pillow which contains an antenna. The PAP recordings are communicated to a clinical hub for data analyzing. The information is obtained when the antenna is held against the body or when the patient lies on the pillow. The entire process is pain free, and patients do not experience any abnormal feeling during the data acquisition process. The electronic unit transmits the PAP measurements daily. The main advantage of CardioMEMS is that the information can be considered by physicians to modify the HF therapies before congestive symptoms present. Based on the obtained information, physicians can promptly adjust the diuretic dose or other HF drugs [41].

The physiopathological basis for the use of CardioMEMS stands on the fact that the symptomatic congestion in HF is typically preceded by a progressive PAP rise [42].

**Figure 5:**
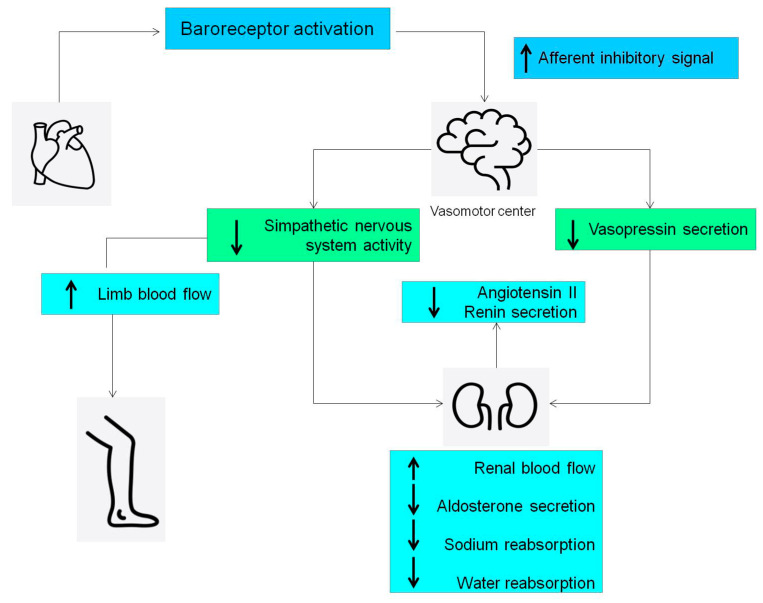
Physiological activation of neurohormonal systems in healthy individuals.

Normally, the cardiopulmonary reflex is activated by the elevation of heart filling pressure (Figure 5).

In healthy individuals, fluid overload activates the pressure. This reflex leads to an increased renal blood flow, inducing sodium and water loss.

This reflex avoids an increase in PAP. In fact, the augmented heart filling pressure, through heart baroreceptors, is transmitted to the vasomotor center in the brain that finally induces an increase in urinary flow [43], restoring the effective circulatory volume and ventricular preload.

**Figure 6:**
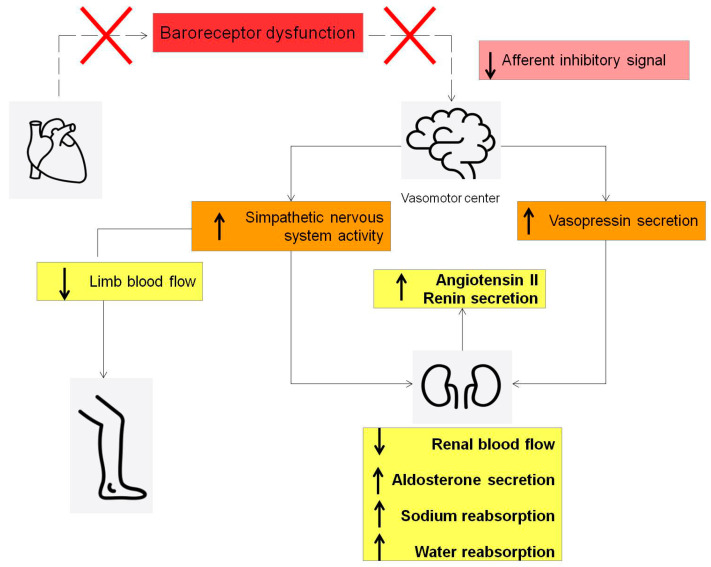
Pathological activation of neurohormonal systems in HFrEF patients.

HFrEF is associated with a significant reduction in the cardiopulmonary reflex [44], leaving a persistent high adrenergic state (Figure 6).

This baroceptor dysfunction induces the brain to activate sympathetic nervous system pathways to target organs and increases the release of vasopressin. The final effect is a reduced sodium and water loss by the kidneys and water retention [42].

The final consequence of this detrimental cascade is a rapid and significant increase in intravascular volume [45], leading to a progressive increase in PAP [42].

CardioMEMS was launched into clinical practice in 2011 by the CHAMPION trial which enrolled 550 patients with chronic HF in NYHA class III with a HF hospitalization within a year prior to enrollment [46]. These patients were randomly divided into two groups: the treatment group which included patients undergoing active monitoring by CardioMEMS, allowing clinicians to use the daily PAP readings on top of OMT, and the control group. The follow-up period lasted 15 months, and patients undergoing CardioMEMS showed a 37% lower risk of HF-related hospitalizations. After this initial phase, PAP data became available for all patients, and patients were then followed for a mean period of 13 months [47]. During this period, there was a significant reduction in HF hospitalizations in the former group compared with the hospitalization rate in the control group [47]. The rate of device-related complications was very low (1%), and the system was proved to be safe. CardioMEMS received FDA approval in 2014 for patients in NYHA class III and with a HF hospital admission in the previous year [46,47]. A subanalysis of the CHAMPION trial [48] revealed that medication changes were more frequently observed in the active monitoring group than in the control group. Diuretics were frequently adjusted in both groups but significantly more often in the active monitoring group as well as vasodilators and other heart failure drugs. This data supported the notion that remote hemodynamic monitoring can significantly impact HF prognosis due to significant changes in drug interventions and reduced HF hospitalization rates [47].

A second randomized clinical trial was the GUIDE-HF trial that was conducted during the COVID-10 pandemic, and the enrollment phase was low. The trial ended with a neutral result [49]. No significant differences in the primary endpoint (all-cause mortality and total HF events defined as HF hospitalizations and urgent HF hospital visits) were detected.

Therefore, considering the previous trial results, the American Heart Guidelines assigned a class of indication 2b for the use of CardioMEMS in selected adult patients with NYHA class III and a history of HF hospitalizations in the previous year or elevated natriuretic peptide levels on maximally tolerated stable doses of medical treatments and optimal device therapy to reduce the risk of subsequent HF hospitalizations [50].

From a European point of view, in 2020, the MEMS-HF study [51], an observational prospective non-randomized study, included patients with chronic HF with NYHA class III and a recent history of HF-related hospitalization. Outcomes included device- or system-related complications, sensor failure, quality of life and clinical endpoints such as the annualized HF rate and all-cause mortality rate and PAP changes from baseline [51]. A total of 234 patients had a CardioMEMS sensor implanted. After 12 months, 98.3% of the patients were free from device- or system-related complications. During the first six months post implant, the HF hospitalization rate decreased by 62%. The reduction over the complete 12-month follow-up period was 66%, which was greater than in the CHAMPION trial. On average, the mean PAP decreased by 3.4 mmHg at 6 months and 5.5 mmHg at 12 months.

The 2021 ESC-HF guidelines stated that devices that involve an invasive assessment of hemodynamic parameters have shown a modest improvement in effort capacity and quality of life. Thus, at the present time, the evidence is considered too low to support specific recommendations for these implantable electrical devices [22].

### 3.2. Cardiac Contractility Modulation

Among HFrEF therapies, a special role is reserved for new cardiac implantable devices [52]. The currently most used device-based therapy for the treatment of HFrEF is cardiac resynchronization therapy (CRT), an instrument capable of leading to an improvement in cardiac performance and prognosis in patients with HFrEF and a wide QRS (mainly > 150 ms) [53]. Unfortunately, only a minority (approximately 30%) of HFrEF patients present a QRS duration above 150 ms [54]; in order to address the needs of patients with a narrow QRS, a new device capable of improving ventricular performance independently from QRS duration named cardiac contractility modulation (CCM; Optimizer Smart^®^ Impulse Dynamics (USA) Inc., Marlton, NJ, USA) has been developed [55].

This device delivers electrical signals in the myocardial refractory period through the placement of two catheters in the right ventricle. The insertion procedure is performed similarly to a normal pacemaker through a cephalic or subclavian access. The implantation is often right-sided since these patients are already carriers of an implantable cardioverter-defibrillator (ICD). Two active fixation leads are fixated to the right ventricular septum 2–3 cm apart from each other and, where an ICD is already implanted, at least 3 cm from the defibrillation lead. The leads are used for sensing ventricular activity and for the bipolar delivery of high-amplitude CCM signals. Active CCM treatment is generally programmed to be daily delivered for at least 7 h per day in one-hour intervals throughout the day. The target treatment is to reach at least a 90% of CCM therapy effective delivery. [56,57,58,59,60,61].

Detailed features of CCM “pharmacodynamics” were discussed in our previous paper [62]. Briefly, CCM induces both early and late effects on heart. The early effects derive by increasing the phosphorylation state of troponin and myosin-binding protein C [63], leading to a positive inotropic effect. In addition, an increase in the phosphorylation state of phospholamban [64] and titin leads to a positive lusitropic effect [58]. The late-onset effects are obtained by reverting maladaptive gene expression [65] involved in the accumulation of dysfunctional fetal proteins [66]. This effect normalizes the expression of key sarcoplasmic reticulum genes by downregulating ryanodine receptor 2, sarco-endoplasmic reticulum Ca^2+^ ATPase and α-MHC. In addition, CCM favors the increase in chaperone transcriptions (such as HSP70), which in turn prevent aggregation and accelerate the detoxification and disaggregation of misfolded proteins [62].

The final effect of these actions is a reverse in left ventricular pathological remodeling, together with an increase in cardiac performance. HFrEF patients treated with CCM experienced an improvement in functional capacity and quality of life and a reduced rate of HF-related hospitalizations [56,59].

Potential candidates for CCM treatment include HFrEF patients with the following criteria [67]:

- LVEF ≥ 25% and ≤45%;
- NYHA class II and III despite optimal medical treatment;
- QRS duration < 130 ms or unresponsive to cardiac resynchronization treatment;
- Left ventricular end-diastolic diameter < 70 mm;
- Low arrhythmic burden (<8900 premature ventricular complexes in 24 h);
- No acute coronary events in the last three months;
- No recent hospitalizations (in the last month);
- Absence of comorbidities conditioning a life expectancy lower than one year.

In conclusion, CCM is a promising alternative for individuals suffering from HFrEF, and its unique benefits of increasing contractile force without the requirement for more oxygen consumption have the potential to become a cornerstone in the management of this disease.

### 3.3. Left Bundle Branch Area Pacing

Left bundle branch block is a conduction defect leading to asynchronous ventricular activation. In HFrEF, it may contribute to systolic dysfunction from a lack of mechanical force due to cellular apoptosis, interstitial fibrosis and adverse remodeling [68]. The current device treatments are biventricular pacing, His-bundle pacing and left bundle branch area pacing (LBBAP). LBBAP represents a more physiological way to pace the conduction system and may represent a tailored electrophysiological strategy for advanced heart failure.

LBBAP refers to the stimulation of the left bundle branch pacing, the left fascicular pacing and the left ventricular septal pacing. The former is a selective stimulation of the left branch before ramification, while the second refers to a direct engagement of one of its fascicles and is thereby divided into left anterior fascicular pacing, left mid-septal fascicular pacing and left posterior fascicular pacing. Lastly, left ventricular septal pacing directly stimulates the septal endocardium of the left ventricle which rapidly carries the impulse to the left branch [69]. In all cases, the ventricular catheter is inserted through the interventricular septum on the right side and reaches the sub endocardium on the opposite side. The target zones are identified by some EKG characteristics indicating the correct positioning of the lead tip [69,70]. Studies show that LBBAP, compared to a conventional biventricular resynchronization strategy, seems to reduce QRS duration and pacing thresholds, while improving the left ventricular end-diastolic diameter, hospitalizations for HF and LVEF [68]. Other positive effects have been shown in a recent study that showed a reduced incidence in ventricular arrhythmic events and atrial fibrillation [71]. Despite the early positive effects, there are some cases where a diffuse conduction system failure may impair the efficacy of LBBAP. For these rare cases, a new combination has been developed: the so-called LBBAP-optimized cardiac resynchronization therapy. With this technique, both an LBBAP and a coronary sinus catheter are implanted, and although limited data are available, a study suggests that, compared to classical LBBAP, there is an improvement in terms of QRS duration. A potential application of this technology is for patients for whom biventricular pacing or LBBAP alone are not successful [72].

LBBAP has some limitations. Firstly, by screwing the lead inside the interventricular septum, there is an intrinsic risk of septal perforation, coronary laceration, and lead dislodgement. Secondly, the benefits of such therapy in ischemic cardiomyopathy are not clear due to the unique characteristics of infracted tissue and the fact that data from long-term applications are still lacking.

### 3.4. Wearable Cardioverter Defibrillators

Conditions characterized by an elevated risk of fatal arrhythmias for a presumed limited time frame represent a contraindication to an ICD implantation. The most common scenario is the case of a new HFrEF diagnosis with a severe reduction in LVEF (i.e., ≤30%), i.e., after extensive myocardial infarction or acute myocarditis. Another issue is that patients with an initial severe reduction in LFEV may undergo a significant increase in LV function after medical treatment, leaving the original ICD indication. In fact, in the PROLONG trial [73] only 38% of patients kept the original indication at the end of 12-month follow-up compared to the 58% observed after three months.

The current ESC guidelines recommend the implantation of an ICD if a severe impairment of LV function persists for ≥3 months on OMT [22]. However, several studies suggested that the optimal duration for the pharmacological treatment to provide a significant improvement in LVEF must be at least 6 months. Thus, during this time frame, there is an increased risk of life-threatening arrhythmic events (roughly 5%) [74]. In these cases, wearable cardioverter defibrillators (WCDs) might represent a valuable bridge. The first multicentric randomized trial conducted by using WCDs was the VEST study [75]. The study was performed in ischemic cardiomyopathy patients, and WCDs did not demonstrate an effective role in reducing the rate of arrhythmic death. However, only 25% of death patients wore a WCD at the time of death, and the daily wearing time in the VEST trial was below 20 h per day. Thus, WCD compliance by patients is a potential issue that impairs its efficacy. In the WEARIT-France study [76], patients wore a WCD for a longer time (23.4 h a day), and the device proved its efficacy and safety, showing that 1.6% of participants experienced at least one appropriate shock.

Finally, the SAVE-ICD study [74] showed that HF of an ischemic etiology was less prone to significant improvements because the scar tissue does not possess a contractile potential and cannot contribute to the overall systolic function. The lower probability of a significant left ventricular improvement after a large anterior myocardial infarction should be considered when a WCD is considered instead of a definitive ICD [22].

WCD also has telemonitoring abilities that allow for checking additional parameters such as physical activity and heart rate, thus providing further information regarding patient condition [77,78]. In conclusion, the current literature reinforces the use of an extensive application of WCDs in clinical practice for patients at risk of ventricular arrhythmias with temporary contraindications to a definitive ICD implantation.

### 3.5. Ultrafiltration for Acute Decompensated HF

Fluid overload is the most common reason for hospitalization in HFrEF patients [79]. Fluid overload manifests as systemic congestion such as pulmonary oedema and swollen legs.

It also leads to changes at the cellular level, causing systemic endothelial dysfunction and an exaggerated inflammatory response that contributes to renal impairment, a reduced absorptive capacity of bowel and hepatic dysfunction [80]. As a result, an early and fast removal of fluid overload constitutes one of the key treatment goals of acute decompensated HF.

Loop diuretics are the first-line treatment for fluid overload.

Unfortunately, loop diuretics lose their efficacy as the disease progresses, determining the so-called diuretic resistance state [81], which is associated with a worse prognosis [81]. The processes behind diuretic resistance are multiple: reduced intestinal absorption, decreased renal blood flow associated with renal venous congestion and neurohormonal activation. In the clinical setting, diuretic resistance results in insufficient congestion relief and a substantial increase in rehospitalization rates [82].

Different strategies can be employed to overcome diuretic resistance such as the up-titration of diuretic dose, intravenous continuous infusion and sequential nephron blockade [83]. Unfortunately, these strategies carry a high rate of failure, especially in the end stage of HFrEF [84].

In this setting, the mechanical removal of fluid overload by extracorporeal ultrafiltration (UF) is a valuable option.

UF enables the removal of isotonic plasma water from the blood by the application of a hydrostatic pressure gradient that is generated by a pump through a semipermeable membrane (hemofilter) [85]. This process leads to the “intra-vascular refill” phenomenon in which the fluid removed from the blood is constantly replaced by fluid absorbed from the third space. The final effect is a negative water balance and finally a gradual fluid overload resolution.

The fluid removal by UF showed some advantages compared with the one achieved by diuretics. First, UF allowed the removal of a greater quantity of sodium compared with hypotonic urine induced by diuretics [86]. Second, by using UF, the clinician can choose the amount of fluid to remove, while it is unpredictable when diuretics are used. This aspect could be of immense importance, especially in patients with labile hemodynamic stability. Lastly, UF does not create neuro-hormonal activation, conversely to diuretics, unless fluid removal exceeds plasma refilling [87] (Table 2).

The main advantage of UF is that it allows a modifiable ultrafiltration rate, ranging from very low to high (up to 500 mL/h) based on patient hemodynamic tolerance [85]. Thus, it can also be carried out in hypotensive patients with minimal hemodynamic impact.

The first observations on UF in the fluid overload of congestive HFrEF patients were developed between 1993 and 2005. However, the first randomized controlled clinical trial was the Ultrafiltration versus Intravenous Diuretics for Patients Hospitalized for Acute Decompensated Heart Failure (UN-LOAD) trial published in 2007. This trial showed that patients treated with UF experienced significantly greater weight loss and decongestion compared to those who were treated with diuretics [88]. Furthermore, the UF group showed a longer rehospitalization-free interval during the 3-month follow-up period.

On the contrary, in the Cardiorenal Rescue Study in Acute Decompensated Heart Failure (CARRESS-HF) trial, patients in the UF group did not achieve greater weight loss [89]. Some issues were raised about the design of the trial since, in the pharmacological therapy arm, the diuretic dose underwent an adjustment based on patient response, while the rate of UF was delivered uniformly at 200 mL/h. Nevertheless, there was not a significant difference in the two groups for mortality during the 2-month follow-up [89].

The Aquapheresis Versus Intravenous Diuretics and Hospitalization for Heart Failure (AVOID-HF) trial [90] compared early adjustable UF therapy and diuretics. Weight loss was greater in the UF group than in the diuretic arm, while the impact of renal function was neutral. Patients in the UF arm showed a hospitalization-free interval greater than that of the patients in the diuretic group within 3 months after discharge.

Several studies and meta-analyses [91] have confirmed the role of UF therapy on a more efficient weight loss and fluid removal compared with diuretic therapy. In addition, UF was shown to be superior in reducing the rate of HF rehospitalization, while there was no significant difference in mortality or incidence of adverse events.

## 4. Conclusions

HFrEF is becoming a frequent condition that significantly impacts both patient quality of life and survival. It also significantly impacts health system resources. In the last decade, new medical treatments and devices have been developed to reduce HF hospitalizations, improve management and reduce the overall rate of mortality.

At the current time, physicians have different drugs that need to be started and up-titrated. Different strategies have been proposed to select which drug should be prescribed first.

Different devices can identify early signs of upcoming HF in asymptomatic patients or treat life-threatening conditions (arrhythmia or fluid retention) for a limited time frame.

This review article gives the readers a comprehensive view of the most recent and important findings that need to be considered when a HFrEF patient is encountered.

## 5. Future Directions

Current approaches to HF are still full of many promising avenues that are being explored and could significantly shape the future of our diagnostic and therapeutic approaches. One notable focus is the investigation of novel pharmacological therapies and biomarkers, offering potential advancements beyond the current standards [92].

Among experimental pharmacological therapies, a class of novel agents targeting cardiac function, such as omecamtiv mecarbil, shows considerable promise [93]. Omecamtiv mecarbil, a selective cardiac myosin activator, enhances myocardial contractility without increasing intracellular calcium levels, offering a unique approach to improving cardiac performance [94]. Early clinical trials have demonstrated its potential to improve symptoms and functional status in HF patients, making it a compelling candidate for phase III trials more directed to hard cardiovascular endpoints [95].

In the realm of biomarkers, emerging candidates like the soluble suppression of tumorigenicity 2 (sST2) and galectin-3 are gaining attention for their prognostic utility in HF [96]. These markers offer insights into the underlying pathophysiology and aid in risk stratification, enabling personalized treatment strategies. Furthermore, ongoing research is exploring the potential of microRNAs as diagnostic and prognostic indicators, offering a glimpse into the molecular mechanisms underlying heart failure progression [97].

While these therapies and biomarkers are not yet endorsed by regulating authorities, their promising results in preclinical and early clinical studies suggest they could play significant roles in shaping the future landscape of HF management within the next five years.

In addition to pharmacological therapies and biomarkers, the integration of artificial intelligence (AI) into HF diagnostic models holds significant promise for the future of HF management. AI-based algorithms, particularly machine learning and deep learning techniques, are being leveraged to analyze complex datasets, including electronic health records, medical imaging and genetic information, to identify patterns and predict heart failure outcomes [98]. These AI-driven diagnostic models have the potential to enhance early detection and risk stratification, ultimately leading to improved clinical outcomes and resource allocation within healthcare systems.

## 6. Limitations

The application of novel drugs and devices for HFrEF patients has some limitations. Firstly, most drugs and devices were used in highly selected patients in clinical trials. In the practical clinical scenario, patients have different comorbidities such as renal failure or infections. In addition, extreme body weights (i.e., sarcopenic or obese patients) have not been tested in clinical trials.

Secondly, most of the new drugs and devices are expensive. A precise selection of patients who may benefit of these options should be considered. As an example, CCM should be not considered in patients with an extremely reduced LVEF (<15%) since the probability of LV recovery is improbable.

Thirdly, although most of the scientific literature that supports ultrafiltration in HFrEF was collected more than 10 years ago, this technique has the potential to become largely used in the current era. In fact, the burden of HFrEF is increasing together with its complexity due to frequent patient comorbidities. We may expect an increasing interest in ultrafiltration in the near future.

## Figures and Tables

**Table 1:** SGLT2 inhibitors approved for HFrEF: main features.

SGLT2-i Molecules	Dosage	Frequency	Contraindications	Side Effects
Dapagliflozin	10 mg	Once daily	Severe reduction in eGFR (eGFR < 25 mL/min/1.73 m^2^) and on dialysis Known hypersensitivity to drug Pregnancy and breastfeeding	Genital fungal and urinary infections Diabetic ketoacidosis Hypotension Hypoglycemia when used with insulin or insulin secretagogues Lower limb and soft tissue infections Dyslipidemia
Empagliflozin	10 mg	Once daily	Severe reduction in eGFR (eGFR < 20 mL/min/1.73 m^2^) and on dialysis Known hypersensitivity to drug Pregnancy and breastfeeding	Genital fungal and urinary infections Diabetic ketoacidosis Hypotension Hypoglycemia when used with insulin or insulin secretagogues Lower limb and soft tissue infections Dyslipidemia

eGFR: estimated glomerular filtration rate.

**Table 2:** Comparative differences between loop diuretics and extracorporeal ultrafiltration.

	Loop Diuretics	Extracorporeal Ultrafiltration
Neurohormonal activation	Present	Absent
Fluid elimination	Hypotonic urine	Isotonic plasma water
Control of fluid and sodium removal	Unpredictable	Precise and efficient
Effect on renal function	Diuretic drugs resistance with continued administration	Possible restoration of diuretic drugs responsiveness
Effect of plasma components	Hypokalemia and hypomagnesemia	None
Anticoagulation	Unnecessary	Necessary
Extracorporeal circuit	Absent	Present
